# Infection risk among adults with down syndrome: a two group series of 101 patients in a tertiary center

**DOI:** 10.1186/s13023-018-0989-x

**Published:** 2019-01-11

**Authors:** Aurélien Guffroy, Yannick Dieudonné, Beatrice Uring-Lambert, Joelle Goetz, Yves Alembik, Anne-Sophie Korganow

**Affiliations:** 1 Department of Clinical Immunology and Internal Medicine, National Reference Center for Autoimmune Diseases, Hôpitaux Universitaires de Strasbourg, 1, place de l’Hôpital, 67091 Strasbourg, France; 2 Department of Immunobiology, Hôpitaux Universitaires de Strasbourg, 67091 Strasbourg, France; 3 Department of Clinical Genetic, Hôpitaux Universitaires de Strasbourg, 67091 Strasbourg, France

**Keywords:** Down syndrome, Immunodeficiency, Infectious risk, Neurological impairment, Adult

## Abstract

**Background:**

Down syndrome (DS) is the most common form of viable chromosomal abnormality. DS is associated with recurrent infections, auto-immunity and malignancies in children. Little is known about immunity and infections in DS at adulthood.

**Methods:**

We studied two separate group of adults (> 18 years old) with DS in a single referral tertiary center (Strasbourg University Hospital). The first group included 37 ambulatory DS patients between November 2014 and May 2017. We analyzed exhaustive serological and immunobiological parameters, at one point, together with the prevalence of infections, autoimmune manifestations and malignancies. The second group included 64 hospitalized patients (138 stays) in the same center, between January 2005 and December 2016.

**Results:**

One hundred and one adult patients with DS were included. Unlike children and despite a global lymphopenia, adults with DS underwent few infections in our ambulatory group. They did not experience any malignancy and, apart from hypothyroidism, they presented only occasional autoimmune manifestations. Hospitalized DS patients were older than ambulatory ones (median age 47 years (18–73) vs. 27 (18–52), *p* < 0.0001) and admitted mostly for infections (76.8%). Infections were associated with epilepsy and dementia (OR 6.5 (2.2–19), *p* = 0.001; *p* = 0.0006 in multivariate analysis) and higher mortality (OR 7.4 (1.4–37), *p* = 0.01).

**Conclusion:**

Despite persistent immunobiological abnormalities at adulthood, young ambulatory adults with DS remain healthy with a low rate of infections. Infections are associated with neurological degeneration and increase the mortality arguing for a dedicated support of older DS patients.

**Trial registration:**

ClinicalTrials.gov: NCT01663675 (August 13, 2012). Hospital Clinical Research Program (PHRC): number 2012-A00466–37 (Dr Y. Alembik).

**Electronic supplementary material:**

The online version of this article (10.1186/s13023-018-0989-x) contains supplementary material, which is available to authorized users.

## Introduction

Down syndrome (DS) caused by trisomy 21 is the most common form of viable chromosome abnormality in children and the prevalence of DS continues to increase with life expectancy [1]. Survival of patients with DS improved drastically in the past few decades, with the detection and the early surgical care of congenital heart malformations (atrioventricular septal, ventricular septal, and atrial septal defects or persistent patent ductus arteriosus) [2]. The median age at death is now mid-50’s compared to 10 years of age in the 1970’s [3, 4]. Children with DS have a high incidence of infections of the respiratory tracts [5]. Over the last 3 decades, these infections have been linked to both innate and adaptive immunological abnormalities. Studies describing the immune system of infants with DS report the reduction and an altered distribution of T and B cell populations [6, 7] coupled to a poor response to vaccines [8–11]. Thus, it has been suggested that children with DS share similarities with patients affected with primary immunodeficiency (PID) and some PIDs classifications include DS [12, 13]. In contrast with pediatric literature, there is a lack of information about infections and immune parameters in adults with DS. Herein we report the features of 101 adults with DS.

## Methods

**Fig. 1:**
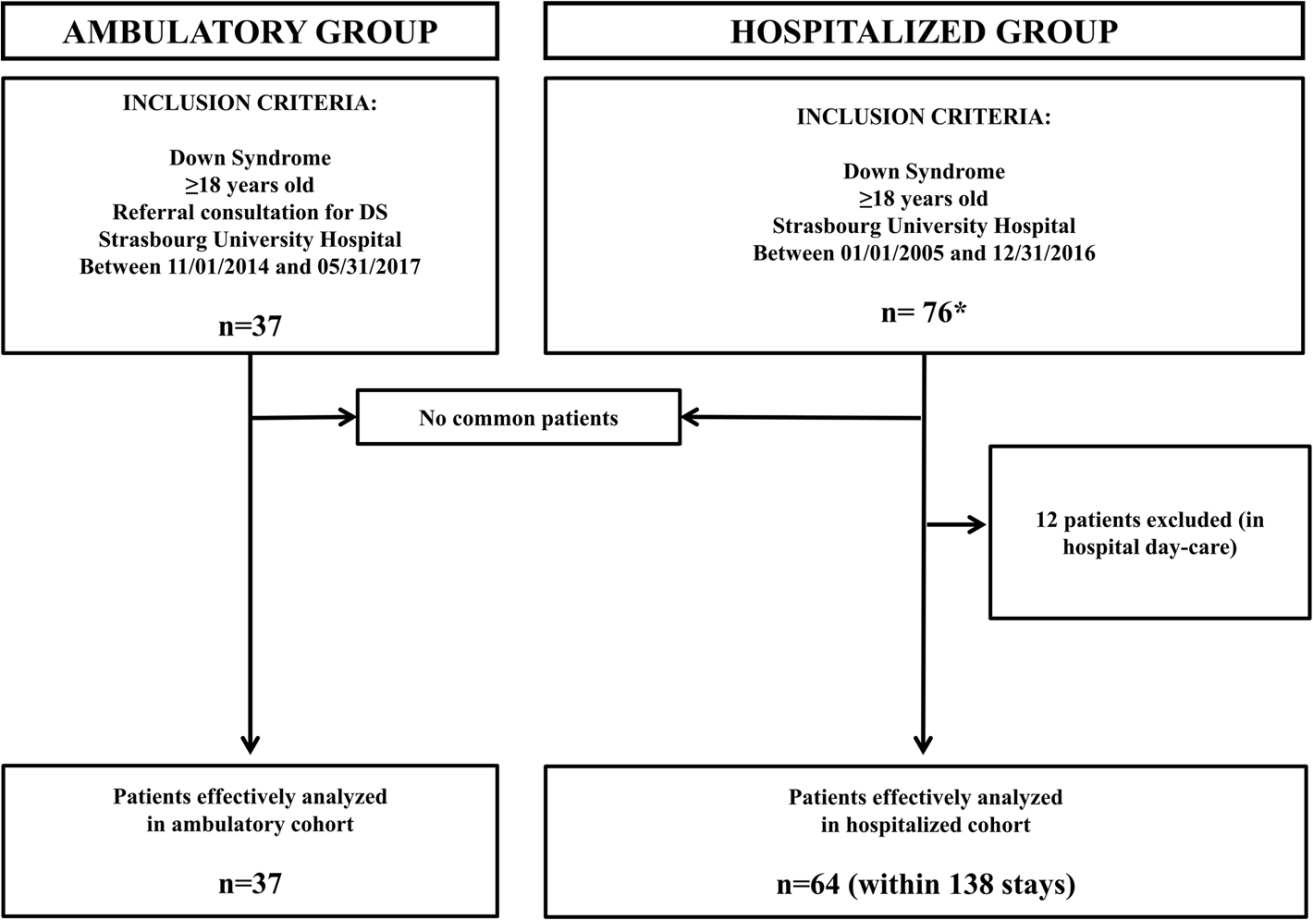
Flow diagram of patients. *Within 190,740 stays - **Patients were excluded for insufficient data when the cause of hospitalization was not mentioned, or when clinical and biological data were unavailable

Following approvement of our Institutional Boards, we studied two separate groups of adults (> 18 years old) with DS in Strasbourg University Hospital (Fig. 1). The first group included ambulatory DS patients (Department of Medical Genetic) between 2014 and 2017. We analyzed at one point serological and immunobiological parameters, together with the retrospective prevalence of infections, autoimmune manifestations and malignancies. The second group included hospitalized patients between 2005 and 2016 with associated DS ICD codes or key words “Down syndrome” or “Trisomy 21” in medical letters with the mean of systematic research by the medical information department of the hospital. We excluded patients hospitalized for scheduled exams.

We used Fisher’s exact test to compare qualitative variables and Student test for quantitative variables in univariate analysis. Mann-Whitney test was used to compare non-normally distributed variables. Multivariate analyzes were done for a *p*-value < 0.10 in univariate analysis using ridge logistic regression. Statistical significance was defined by *p* < 0.05 in 2-tailed tests.

## Results

Thirty-seven patients came to the medical genetic consultation for DS (Table 1). Median age was 27 years (18–52). None was in institutional care. Frequent infections were reported during childhood for 20 DS patients (54%), the leading manifestations involving respiratory tract (49%) and ENT (16%). Only one patient was identified with recurrent infectious events after the age of 18. Total lymphocyte counts of DS patients were low when compared to standards (1480 cells/μL vs. 2170 cells/μL, *p* = 0.004) concerning especially naïve and memory B cell subsets as described in DS children. 15 patients presented with hypergammaglobulinemia (IgG > 15 g/L) and increased IgG1 and IgG3 levels. Serological status following vaccination for DPT (Diphteria, Polio and Tetanus), *Streptococcus pneumoniae* and *Haemophilus influenzae* showed protective titers in most cases.

**Table 1:** Ambulatory DS patients

Demographics and clinical characteristics of ambulatory DS patients		*n* = 37
Age at inclusion (years)		27 (18–52)
Sex ratio (F/M)		1.2 (20/17)
Institutional living (n, %)		0
Recurrent infections within childhood		
*Before 5 years old*		18 (49%)
*From 6 to 18 years old*		17 (46%)
Recurrent respiratory tract infections at adulthood^a^		1 (2.7%)
Hypothyroidism		20 (54%)
Epilepsy		2 (5.1%)
Dementia		0
Cardiac congenital disease		13 (35%)
Urogenital malformation		0
Gastrointestinal malformation		2 (5.1%)
Type 1 diabetes		0
Celiac disease		1 (2.7%)
Hematological malignancy		0
Biological features of ambulatory DS patients	*Normal values*	
Neutrophils (cells/μL)	1800-7900	2225 (1110-4970)
Lymphocytes (cells/μL)	1943-2709	1480 (801–3132)
T cells (cells/μL)	700–1900	1208 (514–2516)
CD4+ T cells (cells/μL)	400–1300	612 (273–1307)
CD8+ T cells (cells/μL)	200–700	474 (117–1365)
Naïve CD45RA+ (% of T-cells)	34–60%	31% (9–65)
Memory CD45RO+ (% of T-cells)	33–65%	65% (32–86)
Regulatory T cells CD3 + CD25 + FoxP3+ (% of CD4+)	2.75–5.0%	2.3% (0.3–5.3)
NK cells CD3-CD56+ (cells/μL)	100–400	231 (53–633)
CD19+ B cells (cells/μL)	169–271	71 (42–264)
Naive CD27- IgD+ (cells/μL)	112–169	50 (4–246)
Transitional CD24 + CD38+ (cells/μL)	2–6	2 (1–8)
Switched memory CD27 + IgD- (cells/μL)	18–40	10 (2–22)
Marginal zone CD27 + IgD+ (cells/μL)	22–54	3 (1–15)
Plasmablast CD27-CD38+ (cells/μL)	1–3	3 (1–13)
CD21lowCD38low (cells/μL)	4–11	2 (1–11)
IgG (g/L)	7.2–14.7	14.2 (10.5–21.2)
IgG1 (g/L)	3.41–10.3	10.1 (5.3–14)
IgG2 (g/L)	2.07–6.59	3.5 (1.4–6.4)
IgG3 (g/L) IgG4 (g/L) IgM (g/L)	0.21–1.45 0.013–1.03 0.48–3.10	1.4 (0.4–3.8) 0.1 (0.1–0.8) 0.7 (0.3–2.9)
IgA (g/L)	1.1–3.6	3.1 (1.6–7.3)
Anti-nuclear antibodies (ANAs) (n, %)	≤ 1/160	6 (16%)
Anti-ds DNA antibodies (anti-ds DNA Abs) (n, %)	< 50 U/ml	0
Anti-thyroperoxidase antibodies^b^ (anti-TPO Abs) (n, %)	<34kU/l	2 (5.4%)
Serological status (protective titer^c^)		n = 37
Tetanus		36 (97%)
Polio		33 (89%)
Diphteria		19 (51%)
*Haemophilus influenzae* type b		30 (81%)
*Streptococcus pneumoniae*		34 (92%)

^a^> 2 pneumoniae or bronchitis with need of antibiotherapy (after 18 years old) ^b^Anti-TPO antibodies were searched for 26 patients ^c^37patients were inoculated against diphteria tetanus and poliomyelitis (DTP vaccine), 35 (97%) against BCG (Bacillus of Calmette and Guerin), 27 (73%) against *Bordetella pertussis*, 8 (22%) against *Haemophilus influenza* type b and 2 (5%) against *Streptococcus pneumoniae*. Protective titers were defined using manufacturers’ values

**Fig. 2:**
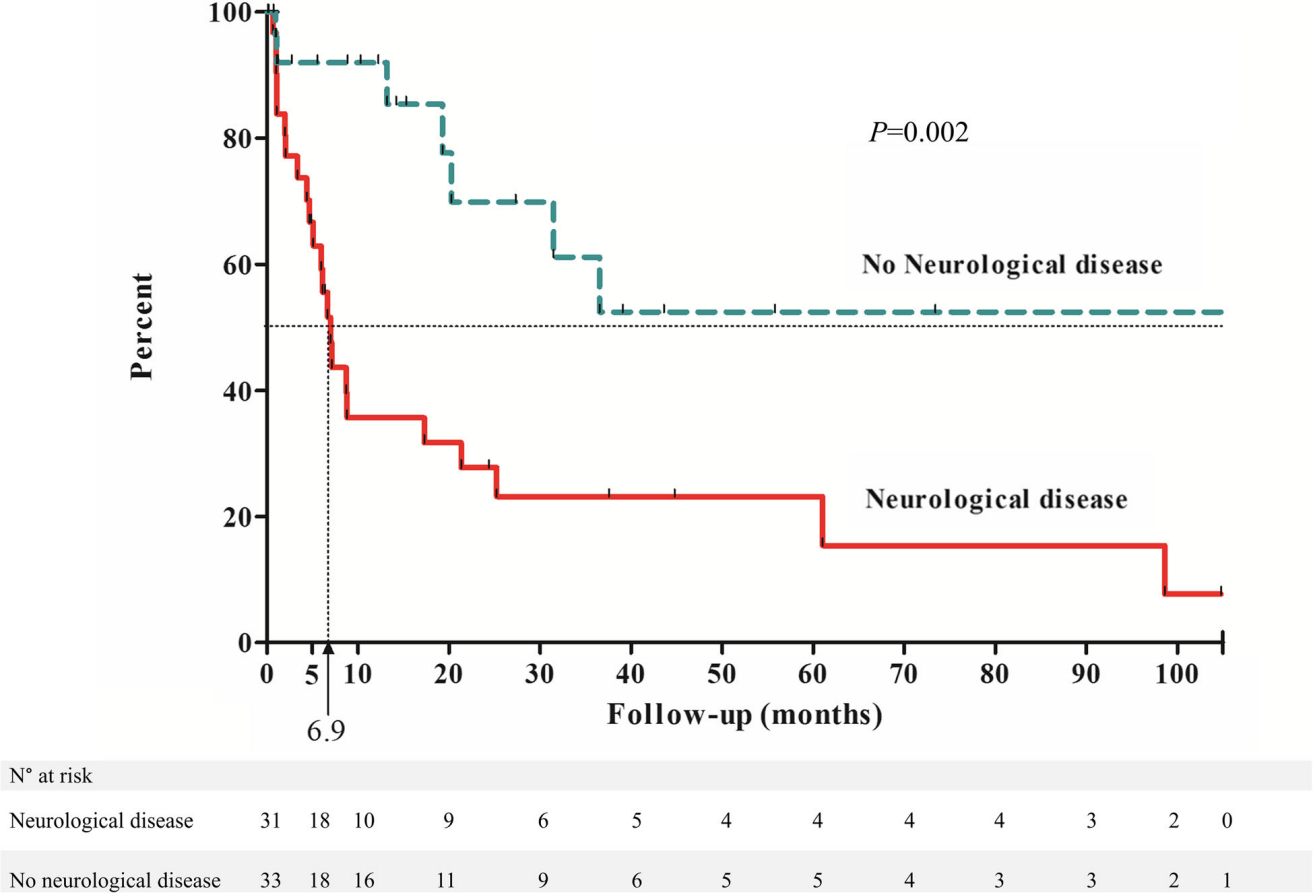
Time to second infectious event within the DS hospitalised group. The median duration of second infectious event-free survival was 6.9 months within the group with neurological disease, as compared with a median over the last follow up (105 months) in the group with no neurological disease. Hazard ratio 0.05 (*p* = 0.002)

During an 11 year period (2005–2016), 64 DS patients were hospitalized at Strasbourg University Hospital, mainly in Internal Medicine, Infectious diseases, Pneumology and Intensive Care Units corresponding to 138 stays (a total of 190,740 stays were recorded in the same departments during this period), (Table 2). Median age was 47 years (18–73), older than ambulatory ones (*p* < 0.0001). Thirty-seven patients (58%) were in institutional care. The outstanding causes for hospitalization among DS patients were infections (*n* = 106/138), mostly aspiration pneumonia (*n* = 91/138). When available, lymphocyte counts and Ig levels were comparable to those of the ambulatory patients. Infections were associated with epilepsy and dementia (OR 6.5 (2.2–19), *p* = 0.001; *p* = 0.0006 in multivariate analysis) and higher mortality (OR 7.4 (1.4–37), *p* = 0.01). We found a median of second infectious event at 6.9 months in the group with neurological diseases vs. more than 120 months in the group without neurological diseases (*p =* 0.002, Fig. 2). Furthermore, the annual rate of infection dramatically increased with age in hospitalized group with a 5 fold increase of incidental infections after 50 years (Additional file 1: Figure S1).

**Table 2:** Hospitalized DS patients (*n* = 64): comparison between DS patients with or without recurrent infections

	Infections^a^ (*n* = 31)	No Infections (*n* = 33)	OR /HR	*Univariate* p*	*Multivariate* p**
Age (years)	51 (19–73)	45 (18–65)	/	**0.01**	0.12
Sex ratio (F/M)	0.35 (8/23)	0.8 (15/18)	0.41 (0.14–1.2)	0.12	0.16
Institutional living (n, %)	21 (68)	16 (48)	2.2 (0.8–6.1)	0.12	0.89
Hypothyroidism (n, %)	14 (45)	13 (40)	1.2 (0.46–3.4)	0.8	/
Cardiac congenital disease (n, %)	5 (16)	5 (15)	1.1 (0.27–4.1)	1	0.06
Gastrointestinal malformation (n, %)	2 (6.4)	2 (6)	1.1 (0.14–8.0)	1	/
Neurological disease^b^(n, %)	22 (71)	9 (27)	6.5 (2.2–19)	**0.001**	**0.0006**
*Epilepsy (n, %)*	16 (52)	7 (21)	3.9 (1.3–12)	**0.02**	/
*Dementia (n, %)*	13 (42)	3 (9)	7.2 (1.8–29)	**0.003**	/
Hematological malignancy (n, %)	0	1 (3)	0.34 (0.1–9)	1	/
Lymphocytes rate (/μL)	1340 (650–2580)	1575 (558–3710)	/	0.09	0.13
Lymphocytes < 1000/mm3 (n, %)	9 (29)	5 (15)	2.3 (0.67–7.8)	0.23	/
Immunoglobulin rate (g/L)	12 (7–23)	16 (9–18)	/	0.22	/
Mortality (n, %)	10 (32)	2 (6)	7.4 (1.4–37)	**0.01**	/

^a^Total number of infectious events: 106 (/138 stays); intensive care unit admission 11/106; Need of pressor amines 7/106, Acute lung injury syndrome 3/18; Pneumonia 96/106 (91%). The other reasons were seizures (*n* = 8), heart failure (n = 5), occlusive syndrome (n = 5), arterial cardiovascular event (*n* = 5), syncope (*n* = 4), acute renal disease (*n* = 2), pancreatitis, venous thrombosis, deep weight loss (n = 1 each)

^b^ Neurological disease was defined as epilepsy or dementia

*Difference between infections and no infections groups using Fischer’s test for categorical variables and Mann-Whitney test for quantitative variables

**Using multiple logistic regression. OR: Odd Ratio – HR: Hazard Ratio

Statistically significant results are marked in boldface

## Discussion

In DS children, epidemiological [1, 14–18] and pathophysiological [6, 19–22] evidences argue for a higher risk of infectious events, hematological malignancies and autoimmunity. Our work correlates for the first time detailed immunological findings and infectious events in adult patients with DS. Despite persistent T and B cell alterations, young ambulatory adults with DS have a low risk of infections, suggesting offsetting mechanisms in adulthood. However, infections, mostly bacterial aspiration pneumonia, remain the first cause of hospitalization. The major factor associated with infectious complications and premature death is the occurrence of neurological diseases such as seizures and dementia [23]. Seizures are frequent in adults with Down’s syndrome with about 10 times increased incidence as compared to general population. Seizures are associated with aging and cognitive impairment in DS [24]. Development of dementia in DS syndrome dramatically increases after age of 40 and is one of the main cause of institutionalization and hospitalization [4, 24]. Considering pediatric studies and our work, infections in DS occur early in life up and in the second adulthood period -after 50 years-old- especially when neurological comorbidities are associated. Indeed, neurological impairment marks a turning point in the infectious complications of adults patients with DS and this should be kept in mind by physicians.

## Additional file

Additional file 1: **Figure S1.** Annual rate of infection by 5-years range within the hospitalized DS patients. (TIF 4139 kb)

## Abbreviations

DPT: Diphtheria, Polio and Tetanus
DS: Down Syndrome
ENT: Ear, Nose and Throat
ICD-10: International Classification of Diseases (version 10)
mAb: monoclonal Antibody
PID: Primary Immune Deficiency
