# Supplementary figures and images for: Infection risk among adults with down syndrome: a two group series of 101 patients in a tertiary center
Source: Orphanet J Rare Dis. 2019 Jan 11;14:15. doi: 10.1186/s13023-018-0989-x (PMC6329099; doi:10.1186/s13023-018-0989-x)

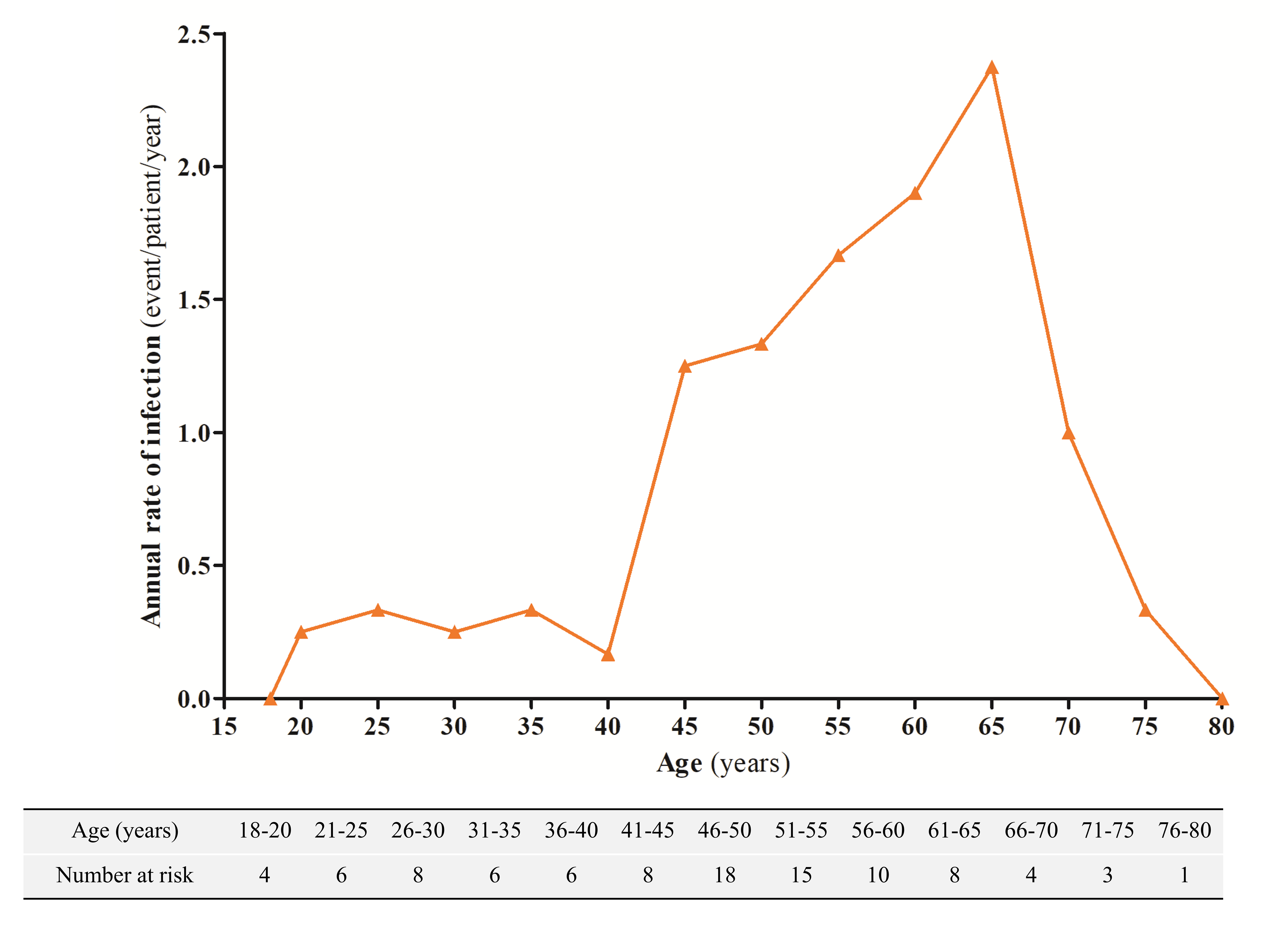

Supplement: Supplementary file 1 — Figure S1. Annual rate of infection by 5-years range within the hospitalized DS patients. (TIF 4139 kb) [file 13023_2018_989_MOESM1_ESM.tif]
